# Un aspect radiologique en aile de papillon ne signifie pas toujours un OAP: pensez à l'adénocarcinome bronchique!

**DOI:** 10.11604/pamj.2014.19.92.5405

**Published:** 2014-09-26

**Authors:** Hicham Janah, Amina Atmane

**Affiliations:** 1 Service de Pneumologie, Hôpital Militaire d'Instruction Mohammed V, Rabat, Maroc

**Keywords:** OAP, adénocarcinome bronchique, dyspnée, APO, bronchial adenocarcinoma, dyspnea

## Image en medicine

Nous rapportons l'observation d'un patient âgé de 58 ans, sans antécédent particulier; se plaignant 1 mois avant son hospitalisation d'un syndrome bronchique, des hémoptysies minimes et une dyspnée d'aggravation progressive devenant une dyspnée au repos évoluant dans un contexte d'apyrexie et d'amaigrissement non chiffré. L'examen clinique trouvait un patient dyspnéique à 28 c/min avec des râles ronflants perçus à l'auscultation pleuro-pulmonaire. La radiographie thoracique montrait un syndrome alvéolaire para-hilaire bilatéral réalisant un aspect en Aile de Papillon (A) faisant évoquer un oedème aigue des poumons. Le scanner thoracique découvrait une collection para-médiastinale droite mesurant 123x110x86 mm à paroi fine et refoulant le médiastin et le parenchyme pulmonaire; avec infiltration tissulaire péri-hilaire et péri-bronchique avec contours externes nets réalisant un aspect en aile de papillon; présence d'adénopathies médiastinales et sous diaphragmatiques (B). La bronchoscopie montrait des éperons épaissis plus marqué au niveau de la lobaire supérieure droite; les orifices rétrécis. Des biopsies bronchiques sur les zones pathologiques ont été faites dont l’étude anatomopathologique et immunohistochimique a conclue à un adénocarcinome moyennement différencie et infiltrant d'origine pulmonaire. Le patient a reçu 6 cycles de chimiothérapie antimitotique. L’évolution est marquée par une stabilité clinique et régression partielle des anomalies radiologiques avec nettoyage de la collection médiastinale (C) sur un recul de 15 mois. L'adénocarcinome pulmonaire primitif représente actuellement le type histologique le plus fréquent du cancer du poumon; de topographie habituellement périphérique mais, d'autres aspects peuvent néanmoins exister.

**Figure 1:**
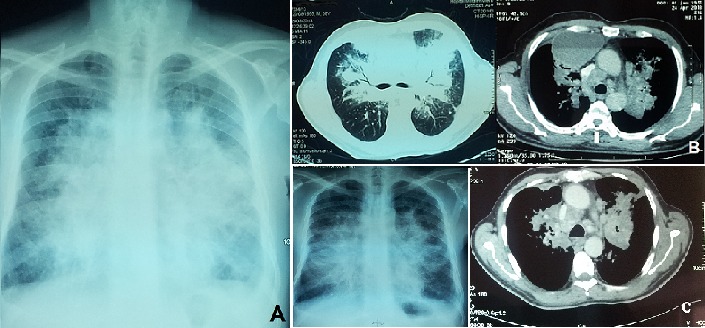
A) radiographie thoracique montrant un syndrome alvéolaire para-hilaire bilatéral réalisant un aspect en aile de papillon; B) scanner thoracique montrant une collection paramédiastinale droite, une infiltration tissulaire péri-hilaire et péri-bronchique en aile de papillon et des adénopathies médiastinales; C) radiographie et scanner thoraciques montrant la régression partielle des lésions après chimiothérapie

